# The effect of microbial transglutaminase and pumpkin peel on physicochemical, microstructural, and oxidative characteristics of camel milk yogurt during cold storage

**DOI:** 10.1016/j.fochx.2025.103355

**Published:** 2025-11-30

**Authors:** Rehab S. Alsulami, Elfadil E. Babiker, Isam A. Mohamed Ahmed, Tawfiq S. Alsulami, Hany M. Yehia, Fahad Y. Al-Juhaimi

**Affiliations:** Department of Food Science and Nutrition, College of Food and Agricultural Sciences, King Saud University, Riyadh 11451, Saudi Arabia

**Keywords:** Pumpkin peel, Rheology, Syneresis, Transglutaminase, Total phenolic, Texture, Yogurt

## Abstract

The influence of MTGase treatment with and without pumpkin peel powder (PPP) on the physicochemical, oxidative, and other quality aspects of camel milk yogurt during refrigerated storage was studied. The MTGase-treated yogurt, with or without PPP, resulted in polymers with a smooth surface and high molecular weight. The total phenolic content (TPC) and antioxidant activity (DPPH) levels increased, while the thiobarbituric acid-reactive substances (TBARS) decreased in yogurt containing PPP during storage. The yogurt treated with MTGase and PPP exhibited significantly higher texture and rheological properties compared to yogurt made with MTGase alone. The yogurt pH decreased during storage, becoming more acidic. The addition of PPP enhanced the water-holding capacity (WHC), lowered syneresis, decreased the L* but increased b*, ΔE, and chroma (C*) values during cold storage*.* Bacterial counts in MTGase-treated yogurt decreased, but were not impacted by the inclusion of PPP during storage. In general, PPP and MTGase improved the overall qualities of yogurt during cold storage.

## Highlights

• Production of camel milk yogurt (CMY) is a challenge due to the low coagulation of CM.
• CMY was developed using microbial transglutaminase (MTGase) and pumpkin peel powder (PPP).
• MTGase and PPP improve the quality parameters of CMY.
• PPP improves the physicochemical properties of CMY.
• PPP improves the oxidative properties of CMY.
• CMY made with MTGase and PPP is a promising product.

## 1 Introduction

Camel milk has long been a healthful substitute for cow's milk in arid areas where other dairy products are scarce. It is also popular because of its unique taste and nutritional benefits (Alia et al., 2023). Camel milk contains healthy lipids, primarily unsaturated fatty acids, which lower the level of cholesterol and prevent plaque from forming in the arteries, as well as reducing lactose intolerance due to its lower lactose content (Swelum et al., 2021). Moreover, it has lysozyme and immunoglobulins, which help fight infections and control the immune system. It is hard to make yogurt and other fermented foods with camel milk because it doesn't coagulate well (Berhe et al., 2017). The yogurt produced is thin, separates more whey, and has an unpleasant texture, which makes it less appealing and less likely to be accepted by customers (Berhe et al., 2017). To solve this problem, scientists have attempted to blend camel milk with milk from other animals to make camel milk yogurt more consistent and less likely to separate (Kamal-Eldin et al., 2020). However, mixing the milk with milk from other animals, on the other hand, changes its natural benefits, properties, and other aspects. Hydrocolloids and stabilizers have been utilized in other studies to increase the consistency of camel milk yogurt. These consist of gelatin (Ho et al., 2022) and a combination of banana fiber and peel fiber (Safdari et al., 2021). Alia et al. (2023) also found that adding gelatin and pulp of persimmon to camel milk yogurt makes it better in terms of its rheological, sensory, and physicochemical aspects.

As a degradable phenomenon affecting the taste and nutritional value of milk, oxidation processes shorten its shelf life and impart an unpleasant aftertaste (Ibrahim et al., 2017). Thus, augmenting the concentrations of physiologically active compounds with antioxidant characteristics to improve milk quality is an essential approach (Stobiecka et al., 2022). The fresh fruit and vegetable industries generate numerous by-products that can be quite profitable, with peels accounting for the most significant portion (Lau et al., 2021). Pumpkin has gained popularity due to its low-calorie content and high fiber, potassium, vitamin A, and vitamin C content (Gavril, Cârlescu, et al., 2024). It also contains a variety of phytochemicals that are beneficial to your health. The fiber in pumpkin helps maintain normal cholesterol levels (AlJahani & Cheikhousman, 2017). The pumpkin peel constitutes approximately 2.6–16 % of the total pumpkin weight, equating to around 0.60–3.68 million tons. It is considered a significant by-product that is typically disposed of in landfills (Nincevic Grassino et al., 2023). Pumpkin peel has been shown to possess significant value due to its content of polysaccharides, carotenoids, minerals, and phenolics. It has potential for recycling and re-utilization as an ingredient in various sectors, including food, cosmetics, and pharmaceuticals (Frosi et al., 2024). According to Lima et al. (2019), pumpkin peel contains a high concentration of carotenoids and pigments that are beneficial to one's health. Pumpkin peel flour contains more fiber and minerals than other flours, and adding it to beef burgers improves their overall taste (Hartmann et al., 2020). Gavril, Stoica, et al., 2024demonstrated that pulp and peels of pumpkin contain numerous phytochemicals, including β-carotene, flavonoids, and phenolics, which improve the immune system and mitigate the effects of aging. Asif et al. (2017) discovered that the methanolic extract of pumpkin peel had increased antioxidant levels.

Protein modification techniques have recently been proposed to enhance the textural and physical characteristics of yogurt using transglutaminase (Lin et al., 2024), protein-glutaminase (Wu et al., 2023), or a combination of transglutaminase and protein-glutaminase (Wu et al., 2024). As described by Wu et al. (2023), yogurt supplemented with MTGase showed greater viscosity and stiffness than untreated yogurt. When compared to the control yogurt, MTGase-treated yogurt was found to be stiffer but less creamy, which decreased its acceptability and preference (Wu et al., 2023). Controlling the cross-linking process is a challenging aspect of utilizing MTGase, as excessive cross-linking can lead to undesirable textural and physical characteristics. Protein-glutaminase catalyzes the deamidation process of glutamine residues in proteins, which improves protein functions, including water solubility, emulsifying qualities, and foaming ability, in contrast to MTGase cross-linking (Sakai et al., 2023). MTGase has recently demonstrated the potential to enhance the qualities of yogurt made from camel milk (Abou-Soliman et al., 2020). Yogurt's three-dimensional structure is stabilized by MTGase cross-linking interactions with milk proteins. It shows improved viscosity and WHC, reduced syneresis, homogeneous structure, desirable texture, and excellent physicochemical stability over time (Gharibzahedi & Chronakis, 2018). Furthermore, they reported that the sensory qualities of yogurt are unaffected by the application of MTGase. One of the best substrates for MTGase among dietary proteins is milk proteins, especially caseins (Bulca et al., 2022). According to Hovjecki et al. (2021), MTGase improved the yogurt's sensory and physical qualities (firmness and whey separation). Similar results were obtained when MTGase was employed in a study by Abou-Soliman et al. (2017), which demonstrated the effectiveness of adding MTGase. Food formulations containing a significant amount of pumpkin peel are expected to exhibit enhanced phytochemical and antioxidant properties. Nonetheless, this may lead to food products exhibiting inferior physicochemical properties and unfavorable sensory characteristics. Consequently, following the adjustment of the incorporated PPP, the impacts of PPP addition and MTGase treatment on the structure, physicochemical and microbiological characteristics, antioxidant capacity, and sensory attributes of camel milk yogurt during cold storage were examined.

## 2 Materials and methods

### 2.1 Materials

In April 2025, fresh pumpkin was obtained from the Riyadh market in Saudi Arabia. To prevent colour loss, the fruits were manually peeled, washed three times with water, and subsequently immersed for five minutes in a 1 % (*w*/*v*) citric acid solution to inhibit colour alteration. The peels were then freeze-dried overnight in a Telstar Lyoalfa-6 Terrassa, Spain freeze-drier. The freeze-dried powder was then stored at −20 °C in sealed polyethylene bags for additional analysis. Standard-grade chemicals such as MTGase (6 U/g milk protein), SDS, M17 agar, MRS agar, gallic acid, DPPH, thiobarbituric acid, BHT were obtained from Sigma-Aldrich (St. Louis, MO, USA).

### 2.2 Set-type yogurt preparation

In preliminary experiments, we faced difficulty in producing control camel milk yogurt without additives or with PPP alone. Therefore, MTGase, as a protein polymerizer, and PPP, which is rich in pectin and polyphenols, were used. Then, the sequence of adding MTGase and PPP was determined. The optimal combination of MTGase and PPP concentrations, along with the MTGase reaction time, was identified as 8 % of MTGase (6 U/g milk protein) with a reaction time of 20 min, and PPP at 1 %. Thereafter, and with a few minor adjustments, the Zhang et al. (2019) method was used to prepare the yogurt. Milk powder was reconstituted in distilled water at a ratio of 1:8 to achieve a total soluble solids concentration of 13 %, comparable to that of fresh milk. The mixture was pasteurized for 30 min at 85 °C, followed by cooling to 24 °C. After adding 1 % (*w*/*v*) PPP to pasteurized milk, the mixture was homogenized for five minutes and heated in a water bath to 55 °C. Then, the sample was mixed with MTGase (6 U/g milk protein) at a concentration of 0.8 % (w/v) and incubated for 20 min. A commercial yogurt culture containing *Lactobacillus delbrueckii* subsp. *bulgaricus* and *Streptococcus thermophilus* (YF-L903, CHR Hansen, Denmark) were then incorporated into the mixture at 43 °C. After that, the mixture was incubated for 4 h at 43 °C to achieve complete coagulation, while maintaining a pH of 4.6. After that, the yogurt was kept for 21 days at 4 °C with a 7-day sample interval.

### 2.3 Gel electrophoresis and scanning electron microscopy (SEM) of set-type yogurt

Utilizing Laemmli's technique (1970), SDS-polyacrylamide gel electrophoresis (SDS-PAGE) was conducted with a 12 % acrylamide separating gel and a 4 % acrylamide stacking gel, each incorporating 0.1 % SDS. Electrophoresis was conducted in a Tris-glycine buffer with 0.1 % SDS, with a current of 10 mA for 1 h, followed by 20 mA for 2 h. Subsequently, the gel sheets were stained with 0.2 % Coomassie Brilliant Blue R-250 and decolorized in a solution of 10 % acetic acid and 20 % methanol for 18 h.

For SEM, the yogurt samples were subjected to a 24-h drying process in a Telstar Lyoalfa-6 freeze-drier located in Terrassa, Spain, for scanning electron microscopy (SEM) analysis. A scanning electron microscope (JEOL JSM-6360 A, Japan) was used to analyze the yogurt powders. The images were captured using the microscope and magnified to 500× and 600×. The samples were coated with a layer 10–12 nm thick for 1.5 min at a working voltage of 20 kV before imaging.

### 2.4 Physicochemical properties of set-type yogurt

A Corning pH meter (Corning Scientific Products, New York, USA) was used to measure the pH levels of the yogurt samples. The titration method was used with 0.1 M NaOH to calculate the acidity of the samples. The acidity of the samples was subsequently determined as a lactic acid percentage. Using a previously published technique reported by Bakry et al. (2019), the samples' syneresis and WHC were assessed. Yogurt samples (10 *g*) underwent centrifugation for 10 min at 5000 ×*g* and 4 °C to facilitate the separation of the drained gel (precipitate) from the supernatant (whey). The precipitate and whey were then weighed independently. The weight of the drained gel relative to the total weight of the yogurt sample (10 g) was known as WHC, whereas the weight of the whey relative to the total weight of the yogurt sample (10 g) was known as syneresis.

### 2.5 Microbiological analysis

A modified method, based on Bakry et al. (2019), was used to examine the samples for bacteria, yeast, and molds after storage for 1, 7, 14, and 21 days. The procedure entailed serial dilutions using peptone water (1:10), initiated by suspending the sample (1 g) in 9 ml of 0.1 g/100 ml sterile peptone water to achieve a dilution of 10^−1^. One milliliter of each dilution was subsequently added to potato dextrose agar (for yeasts and molds), M17 agar (pH 6.5, for *S. thermophilus*), or MRS agar (pH 5.6, for *L. bulgaricus*) plates. The count of *S. thermophilus* and L. *bulgaricus* was conducted by incubating M17 and MRS plates for three days at 45 °C under aerobic and anaerobic conditions, respectively. The plates (PDA) were kept at 37 °C for 5 days to facilitate the count of mold and yeast. The logarithmic values of colony-forming units per gram of yogurt (log CFU/g yogurt) were derived from triplicate analyses.

### 2.6 Yogurt's TPC determination

Using the Folin-Ciocalteu reagent and the technique described by Hernández-Carranza et al. (2016), the TPC in yogurt extract throughout storage was ascertained. A yogurt sample (10 g) was combined with 50 % methanol (80 mL) and shaken for 1.0 h at 24 °C. Following filtration, the samples underwent centrifugation (Shanghai Anting Scientific Instrument Factory, Shanghai, China) at 5000 ×*g* for 5 min at 4 °C to obtain the extract. Folin-Ciocalteu reagent was added, and allowed to stand at room temperature for 5 min. The liquid was then mixed with 1 mL of sodium carbonate (1 mol/L). The mixture was then maintained for an additional two hours at 24 °C. Gallic acid was used to plot a standard curve (0–1.0 mg/ml). A Lambda EZ 150 spectrophotometer (PerkinElmer, USA) was used to determine the absorbance of gallic acid and yogurt extract at 765 nm. TPC was then determined as mg GAE/100 g.

### 2.7 Yogurt's DPPH (2,2-diphenyl-1-picrylhydrazyl) determination

The DPPH of camel milk yogurt extract was measured during storage using the technique outlined by Hernández-Carranza et al. (2016). A DPPH methanolic solution (2 mL, 0.25 mmol/L) was combined with the extract (1 mL), and the mixture was then incubated for 10 min at 24 °C in the dark. A Lambda EZ 150 spectrophotometer from PerkinElmer, USA, was used to measure the absorbance of the samples at 517 nm. The % DPPH was calculated using the following formula:

$$\text{DPPH scavenging}\;\left(\%\right)=\frac{\text{Absorbance of blank}-\text{Absorbance of sample}}{\text{Absorbance of blank}}\times 100$$

### 2.8 Determination of TBARS

The method described by Rosmini et al. (1996) was utilized to determine the amount of TBARS in camel milk yogurt during storage. About 5 g of yogurt and 20 mL ddH2O were combined, well stirred, filtered through paper (Whatman No. 1), and the filtrate was collected to create the yogurt extract. The filtrate (250 μL) was combined with 4 mL of TBA (20 g/mL) and 100 μL of BHT (1 g/10 mL) in a screw-cap tube. The combination was then incubated for 10 min at 95 °C to 100 °C to produce a pink hue. The mixture was then centrifuged at 5500 ×*g* for 25 min, and the supernatant was gathered. At 532 nm, its absorbance was measured. Then, the absorbance was multiplied by 7.8 to compute the malonaldehyde (mg MDA/kg).

### 2.9 Measurements of yogurt texture and rheology

In accordance with Metwalli et al. (2023), the sample's texture profile analysis was conducted utilizing a Brookfield CT3 texture analyzer (Commerce Boulevard, Middleboro, MA, USA), employing an acrylic cylindrical probe with a diameter of 25.4 mm and a length of 35 mm. At a speed of 0.5 mm/s, the probe penetrated the samples to a depth of 15 mm, and automatically recorded the force applied. The qualities noted included hardness, cohesiveness, adhesiveness, and springiness. The formulas employed to calculate chewiness and gumminess are as follows: gumminess is defined as hardness multiplied by cohesiveness, while chewiness is determined as gumminess multiplied by springiness. The samples were analyzed at 4 ± 2 °C for each experiment, and the mean values were recorded. According to Saleh et al. (2020), TA (TA Instruments Inc., New Castle, DE, USA) was used to assess the dynamic viscoelastic characteristics of yogurt samples. The viscosity was measured at a shear rate of 0.62539 1/s, while the storage and loss moduli were measured at an angular frequency of 0.630957 rad/s. Rheology analysis software version 5.7.0 (TA Instruments, New Castle, DE, USA) was used to calculate the findings of three determinations.

### 2.10 Yogurt colour determination

According to Zhang et al. (2019), a CR-300 Minolta colourimeter (Minolta Camera Co., Osaka, Japan) was utilized to assess the colour properties (L*, a*, and b*). A standard white plate was used before colour measurements to calibrate the colourimeter. The colour characteristics of the yogurt samples were evaluated in triplicate using an optically flat glass dish.

### 2.11 Sensory evaluation

Sensory evaluation was conducted in accordance with the guidelines of the Declaration of Helsinki and was approved by the Institutional Review Board (IRB) of King Saud University, Kingdom of Saudi Arabia (Research Project No. E-25-9865). All subjects provided written consent before the study began. Moreover, during the course of the study, acceptable protocols for preserving all participants' rights and privacy were followed. A study team of 37 individuals, comprising both males and females aged 20 to 35, who were either employees or students at King Saud University's College of Food and Agriculture, were invited to evaluate the sensory attributes of the yogurt during its storage. To ascertain sensory qualities, the approach by Dantas et al. (2016) was used. The panelists received three training sessions before the sample inspection to familiarize themselves with the sensory characteristics that would be scrutinized. According to the sensory evaluation, panelists assessed the sensory attributes of the yogurt product while it was being stored. On a 9-point hedonic scale, colour, texture, flavor, sourness, and overall enjoyment were assessed.

### 2.12 Statistical analysis

Three measurements of each parameter and data from a triplicate examination of each yogurt sample were gathered. The analysis was done using IBM SPSS Statistics software, version 23.0 (SPSS Inc., USA), applying a one-way analysis of variance (ANOVA). Duncan's multiple range test was employed to differentiate the means, with *p* ≤ 0.05 considered statistically significant.

## 3 Results and discussion

### 3.1 SDS-PAGE pattern of yogurt

**Fig. 1:**
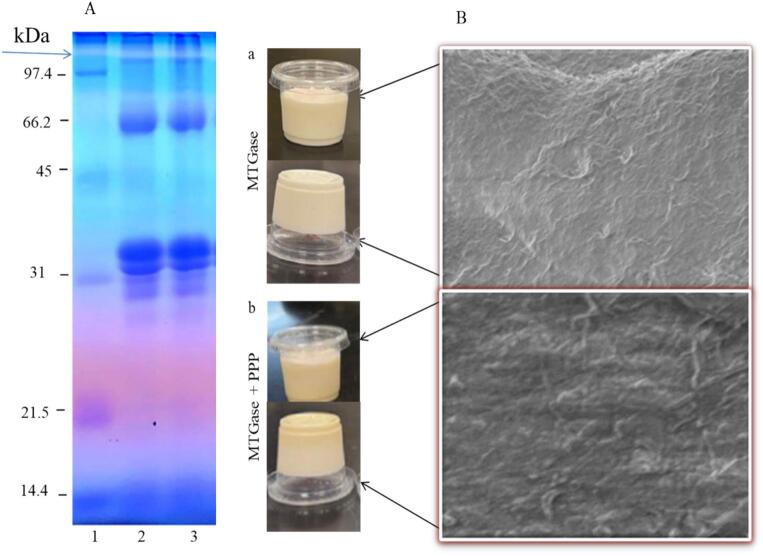
SDS-PAGE pattern (A) of camel milk powder yogurt prepared using microbial transglutaminase (MTGase) with and without pumpkin peel powder (PPP). Lane 1, molecular marker; lane 2, camel milk + MTGase; lane 3, camel milk + MTGase + PPP. Arrows indicate the boundary between stacking (upper) and separating (lower) gels. Scanning electron micrographs (B) of camel milk yogurts fortified with pumpkin peel powder (PPP) and treated with microbial transglutaminase (MTGase). a, MTGase; b, MTGase + PPP.

The impact of MTGase on milk proteins was evaluated through SDS-PAGE analysis (Fig. 1). The polymerization of yogurt protein through MTGase resulted in the emergence of new bands at the upper portion of the separating gel (Fig. 1A, lanes 2 and 3) and a decrease in the monomeric fraction of intact protein in yogurt that has been treated with MTGase and that has been treated with MTGase and PPP samples. Protein molecules experienced cross-linking via a transfer process that involved an amide group in a glutamine-bound protein and an ε-amino group in a lysine side chain, potentially catalyzed by MTGase. The inclusion of PPP in yogurt did not affect the MTGase activity. According to Bulca et al. (2022), who investigated the effect of MTGase on yogurt made from camel milk, their results were consistent with those of the current study. Abou-Soliman et al. (2017) observed that the SDS-PAGE pattern of MTGase-treated yogurt showed a significant reduction in casein intensities and monomeric whey protein bands compared to control samples. The scanning electron microscopy images of yogurt treated with MTGase with or without PPP are shown in Fig. 1B. One day after they were made, the yogurt samples were freeze-dried. The yogurt treated with MTGase, either alone (Fig. 1Ba) or in combination with PPP (Fig. 1Bb), exhibited a denser and more compact structure, a smoother microstructure, and a more homogeneous protein matrix. These alterations likely resulted from the cross-linking of proteins by MTGase, as well as the interaction with pectin and polyphenols of PPP. Bulca et al. (2023) reported that milk proteins, particularly caseins, are the most suitable substrates for MTGase. The results indicate that the protein concentrate serves as an appropriate substrate for MTGase. Therefore, the yogurt exhibited optimal structural distribution and the highest interaction density following treatment with MTGase. The findings indicated that PPP did not influence the activity of MTGase. A study by Bulca et al. (2022) demonstrated that protein matrices in MTGase-treated camel milk yogurt exhibited greater compactness, which was attributed to the influence of MTGase on the microstructure, resulting in higher aggregate density and facilitating the formation of a finer mesh network. A study indicates that incorporating whey protein concentrates and beta-lactoglobulin into yogurt, in conjunction with MTGase treatment, produces a microstructure characterized by homogeneity, smaller clusters, and a more robust network compared to untreated samples (Abou-Soliman et al., 2017).

### 3.2 Physicochemical and microbiological attributes of yogurts

**Fig. 2:**
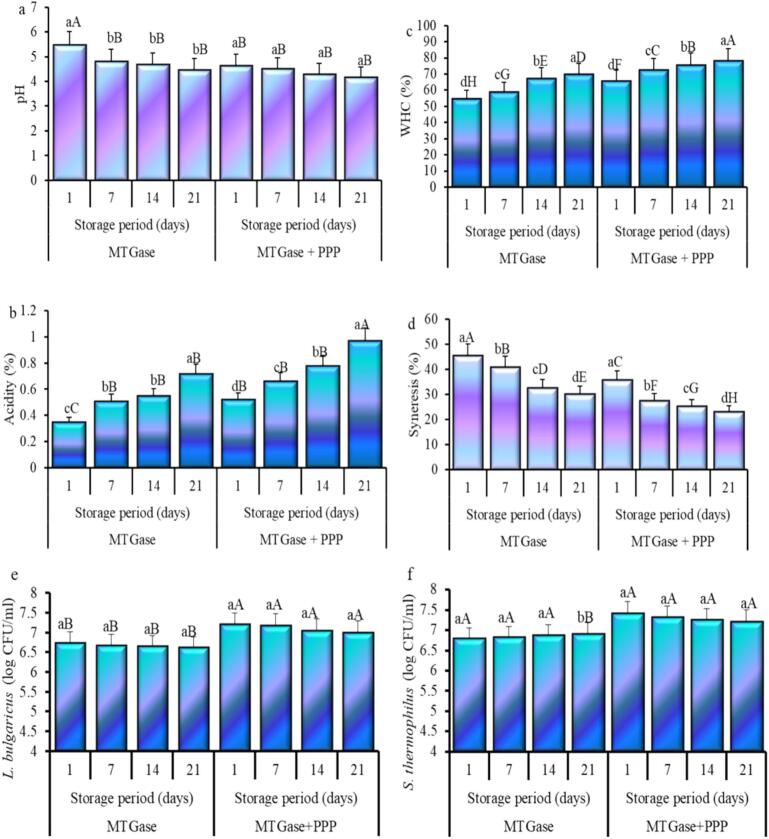
pH (a), acidity (b), water holding capacity (c), syneresis (d), *Lactobacillus bulgaricus* (e), and *Streptococcus thermophilus* (f) of camel skim milk yogurt during storage prepared using microbial transglutaminase (MTGase) with and without pumpkin peel powder (PPP). Small letters denote significant difference within treatment (MTGase or MTGase + PPP), and big letters denote significant difference (*P* ≤ 0.05) between all treatments.

Fig. 2 illustrates yogurt pH, acidity, WHC, syneresis, and microbial analysis after treatment with MTGase with or without PPP. After 7 days, the pH (Fig. 2a) of yogurt treated with MTGase reduced significantly (*P* ≤ 0.05) from its initial elevation, and thereafter it stabilized; however, adding PPP caused a further drop in pH. The pH steadily declined with prolonged storage duration, ultimately reaching a minimum value of 4.16 for MTGase with PPP yogurt after storage. The most notable drop in pH was noticed with MTGase in PPP yogurt. Adding PPP caused a slight drop in pH, from 4.63 to 4.16, in yogurt throughout storage, suggesting that PPP effectively modulates the metabolic activity of lactic acid bacteria (LAB), thereby maintaining the bacterial colony count during the storage period. Comparable results have been documented in yogurt containing jujube pulp (Feng et al., 2019) and extract from date palm spikelets (Almusallam, Ahmed, et al., 2021). Throughout the storage period, the acidity of MTGase-yogurt with PPP increased consistently, peaking at 0.97 % at the end of 21 days (Fig. 2b). The observed pH drop and corresponding increase in acidity during storage are likely due to enhanced metabolic activity of the LAB. These bacteria metabolize lactose, resulting in the production of lactic acid, and further degrade both fiber and fatty acids into uronic acids, which collectively contribute to the drop in pH and increase in acidity. Yogurt samples with date palm spikelets extract (Almusallam, Mohamed Ahmed, et al., 2021), extract from *Moringa* leaf (Zhang et al., 2019), and pulp of jujube (Feng et al., 2019) exhibited comparable alterations in pH and acidity throughout storage.

WHC and syneresis are crucial physical properties of yogurt, as they significantly impact the product's shelf life and acceptability (Arab et al., 2023). This study demonstrates that WHC and syneresis were affected by MTGase treatment, both with and without PPP (Fig. 2c and d). Yogurt treated with MTGase and fortified with PPP exhibited a significantly higher WHC of 77.96 % and a lower syneresis value of 23.04 % compared to yogurt treated with MTGase alone, which showed WHC and syneresis values of 69.82 % and 30.18 % respectively, at the end of storage. MTGase-treated yogurt WHC significantly increased during storage from 54.51 % to 69.82 % while that treated with MTGase and fortified with PPP increased from 65.8 % to 77.96 %. The yogurt syneresis was significantly decreased from 45.49 % to 30.18 % for yogurt treated with MTGase, while for that treated with MTGase and fortified with PPP, it decreased from 35.86 % to 23.04 %. The enhancement in WHC and reduction in syneresis of MTGase-treated yogurt can be attributed to the denser aggregates formed by MTGase, as well as pectin and polyphenols of PPP, which also participate in network formation, contributing to a finer mesh network that increases gel hardness, resulting in smaller pores and decreased syneresis (Gharibzahedi & Chronakis, 2018). The enhancement of WHC and reduction in syneresis following the incorporation of PPP can be attributed to the gelling properties of pectin sourced from pumpkin peels, which demonstrates efficacy for diverse applications (Gavril et al., 2024b). Moreover, the enhanced WHC of yogurt after the addition of PPP can be ascribed to the interaction between milk protein and PPP polyphenols. This interaction may alter the structure and affinity of the protein, resulting in the development of a strong network that can retain higher levels of whey (Kwon et al., 2019). Similarly, fortification with extract from *Moringa* leaf (Zhang et al., 2019) and chia seed (Kwon et al., 2019) has demonstrated improvements in water-holding capacity and reductions in syneresis relative to control yogurts.

Fig. 2 illustrates the L. *bulgaricus* (e) and *S. thermophilus* (f) load (log CFU/ml) of camel milk yogurt during storage, prepared with MTGase with or without PPP. Hygienically, all samples had no coliforms, yeast, or molds throughout the storage time (data not shown). MTGase resulted in a minor reduction in L. *bulgaricus* count, decreasing from 6.75 to 6.63 log CFU/ml (Fig. 2e), but slightly increased from 6.79 to 6.91 log CFU/ml (Fig. 2f) for *S. thermophilus* at the end of storage (21 days). However, MTGase-treated yogurt with PPP exhibited a slight reduction in microbial counts, with L. *bulgaricus* decreasing from 7.21 to 7.01 log CFU/ml (Fig. 2e) and *S. thermophilus* decreasing from 7.42 to 7.21 log CFU/ml (Fig. 2f) at the end of storage. Adding PPP to yogurt enhanced both strains viable counts relative to the MTGase-treated sample; nonetheless, this enhancement diminished slightly over storage, due to the phenolic compounds in PPP that inhibit LAB growth by damaging the cytoplasmic membrane, nucleic acid synthesis inhibition, disruption of metabolic energy, and interference with the activity of enzymes, which encompasses reductions in the integrity of cell wall and synthesis of protein (Jeong et al., 2018). The increase in *S. thermophilus* count was associated with an increase in the titratable acidity of yogurts during storage, indicating that the growth of *S. thermophilus* played a role in the acidity enhancement and demonstrated its ability to adapt to adverse growth conditions related to a drop in pH, as reported by Turgut and Diler (2025). Initially, the viable counts of the two strains in the samples were identical; however, significant variations developed over time. The viable counts of both strains in the MTGase-yogurt sample decreased from day 1 to day 7, ultimately reaching their lowest values by the end of the 21-day storage period. The slight decrease in both strains' viable counts during the first week can be attributed to changes in bacterial metabolic activity, which is affected by variations in pH and acidity levels. The long duration may have inhibited the growth of LAB due to low pH and acidity (Jaster et al., 2018). Notably significant variations (*p* ≤ 0.05) in bacterial counts for both strains were observed between the MTGase-yogurt and the one treated with PPP at the end of the period. Yogurt treated with PPP maintained both LABs viability during storage, indicating health benefits associated with the yogurt. The favourable effect of PPP on maintaining viable LAB counts over 21 days of storage is attributed to the antioxidant compounds found in PPP, which influence LAB metabolic activity in yogurt enriched with PPP. Studies have indicated that adding extracts from Moringa leaves (Zhang et al., 2019) or date palm spikelets (Almusallam, Mohamed Ahmed, et al., 2021) improves the growth of LAB in yogurt. However, a study demonstrates that the inclusion of pulp from jujube (Feng et al., 2019) prevents the growth of lactic acid bacteria in yogurt. The results of this research indicate that the use of PPP with MTGase in yogurt creates suitable environmental conditions for LAB during extended storage.

### 3.3 Oxidative characteristics of yogurt

**Fig. 3:**
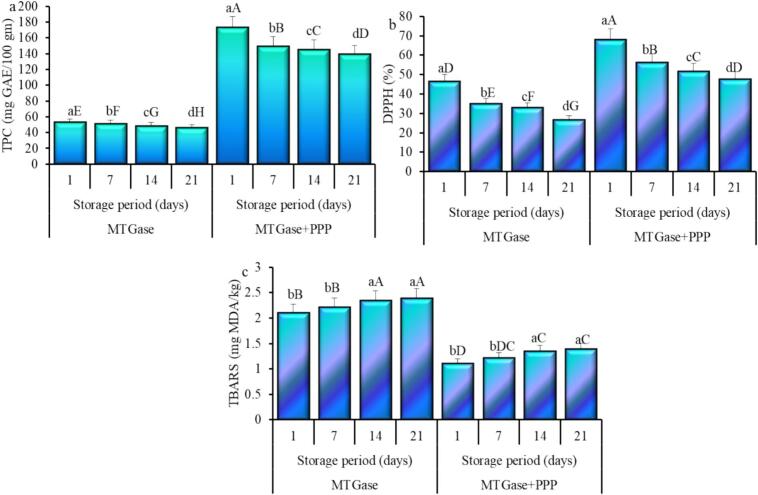
Total phenolics (TPC, a), DPPH radical scavenging activity (b) and thiobarbituric acid reactive substances (TBARS, c) of camel milk powder yogurt during storage prepared using microbial transglutaminase (MTGase) with and without pumpkin peel powder (PPP). Small letters denote significant difference within treatment (MTGase or MTGase + PPP), and big letters denote significant difference (P ≤ 0.05) between all treatments.

The analysis of PPP revealed a TPC of 387.87 mg GAE/100 g and a DPPH activity of 83.89 % (data not shown). Fig. 3 illustrates the TPC, DPPH, and TBARS levels in camel milk yogurt subjected to MTGase treatment, both with and without PPP, during cold storage. The TPC of yogurt treated with MTGase was measured at 53.15 mg GAE per 100 g, which corresponds to a DPPH value of 46.39 %. A slight decrease was noted during the storage period, culminating in a minimum TPC of 46.27 mg GAE/100 g, accompanied by a significant (*P* ≤ 0.05) reduction in DPPH value to 26.75 % by the end of the storage period (Fig. 3b). The incorporation of PPP into treated yogurt led to a notable increase (P ≤ 0.05) in TPC to 173.49 mg GAE/100 g, alongside a DPPH value of 68.16 % (Fig. 3b). During the storage period, the TPC decreased significantly (P ≤ 0.05) to 139.25 GAE/100 g, accompanied by a notable (P ≤ 0.05) decline in DPPH to 47.76 %. The results indicate that the addition of PPP to yogurt significantly (p ≤ 0.05) enhanced TPC levels and elevated DPPH activity. Research indicates that PPP shows markedly elevated levels of TPC and antioxidant activity (Zaini et al., 2022). The increased TPC in PPP accounts for the rise in TPC and DPPH levels in yogurt upon the addition of PPP. Alenisan et al. (2017) conducted an investigation demonstrating that increased levels of natural antioxidants significantly enhanced total phenolic content (TPC) and DPPH radical scavenging activity in yogurt. During storage, the total phenolic content (TPC) decreased, which was associated with a reduction in DPPH activity. The decrease in TPC and DPPH levels in yogurt during cold storage may be attributed to interactions with other ingredients that mitigate oxidative rancidity (Caipo et al., 2021). Pascariu et al. (2025) observed a significant initial total phenolic content that decreased with prolonged storage time, linking this decline to the gradual degradation of polyphenolic compounds. The increase was due to the degradation of milk proteins by proteases, resulting in the release of amino acids with phenolic side chains. The authors proposed that microbial metabolism could have facilitated the formation of novel phenolic acids, consequently elevating the total levels of polyphenols.

The effect of treatment on TBARS (Fig. 3c) showed that adding PPP with MTGase to yogurt lowered lipid peroxidation. The MTGase-treated yogurt and that treated with MTGase and PPP initially showed low values of TBARS. However, the results of TBARS of the samples treated with MTGase exhibited a significant increase throughout the storage period, resulting in a peak of 2.39 mg MDA/kg on the final day of storage. The yogurt formulated with MTGase-PPP exhibited a maximum TBARS value of 1.39 mg MDA/kg following storage. The findings demonstrate that PPP enhances the lipid oxidation stability of yogurt in comparison to yogurt formulated with MTGase. The notable rise in TBARS values of MTGase-yogurt and the modest increase in TBARS values of MTGase-yogurt supplemented with PPP during storage indicate lipid oxidation and aldehyde production in the yogurt (Almusallam et al., 2021a). The antioxidant constituents in PPP-fortified yogurt enhanced storage stability and prolonged the shelf life compared to yogurt treated solely with MTGase. Research demonstrates that the addition of Argel leaf extract (Mohamed Ahmed et al., 2021) and date palm spikelet extract (Almusallam et al., 2021a) to yogurt led to decreased TBARS levels and improved stability of the fortified yogurts under refrigeration.

### 3.4 Texture and rheology of yogurt

Yogurt texture evaluation is a critical indicator of quality and significantly influences consumer perception and satisfaction. The texture profile analysis of the yogurt samples is presented in Table 1. Adding PPP and MTGase treatment leads to a significant (*p* ≤ 0.05) enhancement in yogurt hardness, elevating it to 27.12 g compared to that of yogurt treated with MTGase, which resulted in 20.45 g of hardness at the end of storage. Hardness is an essential textural characteristic of yogurt. The increased firmness observed in PPP-incorporated yogurt during storage is due to a greater degree of protein rearrangement relative to yogurt that has not been treated with PPP. The enhanced hardness of PPP-fortified yogurt is due to the improved WHC within the gel system. Additionally, increased protein ratios result in a denser and more stable yogurt structure. Contrary to our findings, an earlier study demonstrated that the addition of jujube pulp (Feng et al., 2019) to yogurt resulted in reduced firmness compared to control samples. Covalent cross-linking reactions by MTGase induce notable alterations in size, conformation, stability, and other properties, as observed by Wu et al. (2023), who showed that the incorporation of MTGase improved textural and sensory characteristics and decreased syneresis during storage. Also, Abou-Soliman et al. (2017) found that the application of MTGase to camel milk yogurt enhanced its hardness. During the storage period, the hardness of the MTGase-treated yogurt increased from 19.51 to 20.45 g, whereas the hardness of the PPP-fortified yogurt treated with MTGase increased from 21.75 to 27.12 g. The incorporation of PPP with MTGase significantly enhanced the hardness of yogurt during storage compared to MTGase alone. The hardness of yogurt is influenced by several factors, including the composition of the starter culture, total solids, protein content, and type, as well as the interactions among these components (Arab et al., 2023). The cohesiveness, springiness, adhesiveness, gumminess, and chewiness were greater in PPP-fortified and MTGase-treated yogurts compared to those treated with MTGase alone, indicating that the combination of PPP and MTGase improved the textural properties of yogurt. The observed improvement in these parameters after adding PPP can be explained by the fact that the protein matrix has high WHC, the dominance of protein in the yogurt formulation, and the characteristics of the protein matrix itself. The parameters of the yogurt increased during storage from day 1 to day 21, likely because storage induces various chemical reactions that are anticipated to disrupt the casein strands within the gel network. This disruption results in a decrease in casein micelle aggregates, an increase in protein rearrangements, and a reduction in the stability of the casein network (Pan et al., 2019). Nonetheless, the presence of bioactive substances (Pan et al., 2019) and MTGase treatment (Abou-Soliman et al., 2017) can create protein complexes containing amino acid side chains in the gel. This interaction can help close gaps in the protein matrix, stabilizing the casein network and thereby enhancing the smoothness of yogurt. This theory explains the observed changes in textural profile after storage of treated yogurts. Pomace from apples (Wang et al., 2020) and powder from pomegranate juice (Pan et al., 2019) have been shown in studies to yield comparable outcomes when added to yogurt.

**Table 1:** Texture of camel skim milk yogurt during storage, prepared using microbial transglutaminase (MTGase) with and without PPP.

Treatment/ Storage period (days)	Texture					
	Hardness (g)	Cohesiveness	Springiness (mm)	Adhesiveness (mj)	Gumminess	Chewiness
*MTGase*						
1	19.51 ± 0.44^bF^	0.48 ± 0.06^bA^	7.02 ± 0.48^aB^	0.26 ± 0.02^bA^	9.36 ± 1.02^bD^	65.74 ± 1.78^dH^
7	19.52 ± 0.29^bF^	0.51 ± 0.05^abA^	8.11 ± 0.54^aB^	0.29 ± 0.03^bA^	9.96 ± 0.81^bCD^	80.74 ± 1.21^cG^
14	19.95 ± 0.51^baF^	0.52 ± 0.02^abA^	8.21 ± 0.45^aB^	0.31 ± 0.04^abA^	10.37 ± 0.46^bC^	85.17 ± 1.22^bF^
21	20.45 ± 0.62^aE^	0.62 ± 0.04^aA^	8.91 ± 0.47^aB^	0.32 ± 0.07^aA^	12.68 ± 0.47^aB^	112.97 ± 0.98^aC^
*MTGase + PPP*						
1	21.75 ± 0.67^dD^	0.46 ± 0.13^aA^	9.17 ± 0.37^bA^	0.28 ± 0.07^bA^	10.01 ± 0.58^dC^	91.75 ± 1.35^dE^
7	23.32 ± 0.57^cC^	0.48 ± 0.11^aA^	9.43 ± 0.67^aA^	0.31 ± 0.08^bA^	11.19 ± 0.77^cC^	105.56 ± 1.49^cD^
14	25.15 ± 0.67^bB^	0.51 ± 0.07^aA^	9.78 ± 0.72^aA^	0.37 ± 0.06^abA^	12.83 ± 0.79^bB^	125.44 ± 1.11^bB^
21	27.12 ± 0.89^aA^	0.68 ± 0.06^aA^	9.79 ± 0.45^aA^	0.39 ± 0.05^aA^	18.44 ± 0.47^aA^	180.54 ± 1.41^aA^

Values are means of three samples ± SD. Small letters denote significant difference within treatment and big letters denote significant difference (P ≤ 0.05) between treatments.

**Fig. 4:**
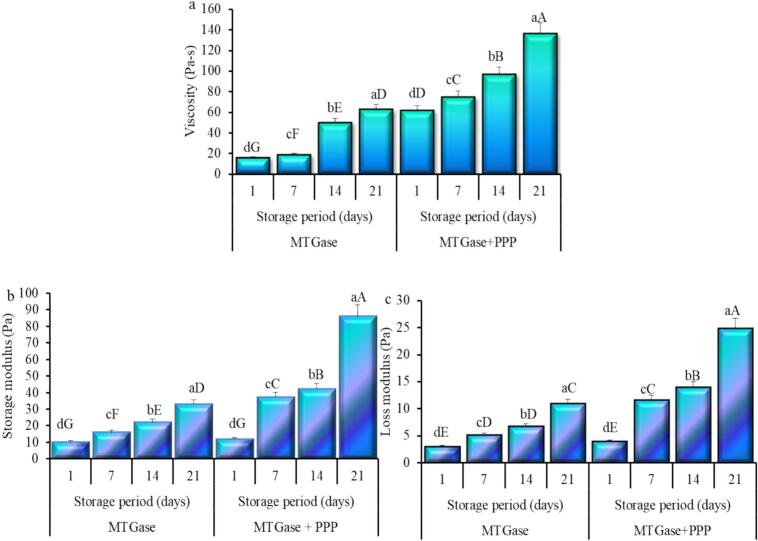
Viscosity (a), storage modulus (b), and loss modulus (c) of camel milk powder yogurt during storage prepared using microbial transglutaminase (MTGase) with and without pumpkin peel powder (PPP). Small letters denote significant difference within treatment (MTGase or MTGase + PPP), and big letters denote significant difference (P ≤ 0.05) between all treatments.

The rheological characteristics, specifically viscosity, storage modulus, and loss modulus, of camel milk yogurt treated with MTGase with or without PPP were evaluated and are presented in Fig. 4. The findings indicated a significant (*P* ≤ 0.05) increase in viscosity (Fig. 4a) within and between treatments of yogurt samples. The viscosity increased from 15.61 Pa-s after 4 h of incubation to 62.58 Pa-s at the end of storage time for MTGase-treated yogurt, whereas for yogurt treated with MTGase and PPP, it increased from 61.71 Pa-s after 4 h of incubation to 136.23 Pa-s at the end of storage time. Abou-Soliman et al. (2017) found that milk fortification with MTGase enhanced yogurt viscosity. The viscosity of yogurt treated with MTGase and fortified with PPP was significantly elevated due to compositional variations, particularly in protein content, compared to unfortified yogurt. This increase in protein content, as well as the presence of pectin and polyphenols in PPP expected to enhance the extent of polymerization and the yogurt's apparent viscosity. The Low values in viscosity observed after MTGase treatment, in contrast to PPP, are likely attributable to protein cross-linking, which diminishes the proteins' ability to bind water. Moreover, variations in formulation, types of cultures, thermal processing conditions, and methods applied may significantly influence yogurt viscosity (Arab et al., 2023). The high viscosity of the samples during storage can be attributed to the formation of a firm gel, which resisted degradation by lactic acid bacteria, resulting in diminished gel stability and increased fluid release (Yang et al., 2023). Moreover, the observed increase in viscosity over storage time in PPP-fortified yogurts compared to MTGase-treated ones can be attributed to post-acidification occurring at 4 °C, where the proteins in milk contribute to the production of a firmer gel, resulting in enhanced yogurt viscosity (Mohammadi-Gouraji et al., 2019). In addition, the production of exopolysaccharides by LAB under adverse storage conditions, as reported by Parvarei et al. (2021), may contribute to the increased viscosity observed in PPP-fortified yogurts. An increase in yogurt viscosity enriched with phycocyanin has been documented (Mohammadi-Gouraji et al., 2019). Also, an increase in yogurt viscosity enriched with pomace from grapes has been documented during cold storage (Demirkol & Tarakci, 2018).

As shown in Fig. 4, both storage (Fig. 4b) and loss (Fig. 4c) moduli increased with the storage period, exhibiting significant variation between and within treatments of yogurt. The storage modulus increased from 10.04 Pa after 4 h of incubation to 33.09 Pa at the end of storage time for MTGase-treated yogurt. For yogurt treated with MTGase and PPP, the storage modulus increased from 11.89 Pa after 4 h of incubation to 85.94 Pa at the end of storage time. Additionally, the loss modulus increased from 2.99 Pa after 4 h of incubation to 10.89 Pa at the end of storage time for MTGase-treated yogurt. In contrast, for yogurt treated with MTGase and PPP, the loss modulus increased from 3.92 Pa after 4 h of incubation to 24.77 Pa at the end of storage time. The PPP-treated yogurt had significantly higher values of such moduli compared to that treated with MTGase alone. All samples exhibited a storage modulus that was greater than the loss modulus throughout the storage period. The elevated storage modulus values relative to the loss modulus suggest that all experimental yogurts exhibited greater elastic than viscous properties, indicating a maximum gelation capability of the produced yogurt. The findings indicated that extending the storage period significantly enhanced the viscoelastic properties of the samples. The data indicate that incorporating PPP into yogurt treated with MTGase improves the elastic characteristics of the yogurt. This can be explained by the observation that casein micelles were primarily connected through particle-to-particle attachment in extensive chains featuring relatively small interspersed cavities rather than through particle fusion into aggregates (Shi, Han, & Zhao, 2017). Research indicates that the pretreatment of milk with TGase results in an increase in the apparent viscosity of yogurt samples (Hovjecki et al., 2021), as also observed by Zhang et al. (2021), who stated that the cross-linking of whey protein induced by TGase can enhance the apparent viscosity, as well as its storage and loss moduli values.

### 3.5 Colour characteristics of yogurt

The colour of yogurt is a significant attribute that influences consumer acceptance and marketability (Turgut & Diler, 2025). The colour parameters (L*, a*, and b*) varied between yogurt treated with MTGase and PPP-fortified yogurts (Table 2). Adding PPP to yogurt resulted in a significant decrease in the values of both L* and a*, alongside an increase in the b* value, when compared to yogurt treated with MTGase. The change in colour attributes suggests that PPP diminished the lightness and greenness while enhancing the yellowness of the yogurt. The chroma (C*) exhibited significant variation between yogurt treated with MTGase and that treated with MTGase and enriched with PPP, with the latter displaying higher values. On the first day and the rest of the storage period, the highest L* values were recorded in yogurt treated solely with MTGase, while the highest b* values were found in yogurt treated with MTGase and supplemented with PPP. The a* values between the two treatments were significantly different (*P* ≤ 0.05). This may be attributed to the yellow hue of PPP. The L*, a*, and b* values of all yogurts exhibited fluctuations during storage, as reported by Gavril et al. (2024b), who developed a value-added yogurt using PPP as a bioactive powder. The colour change (ΔE) following the addition of PPP was initially minimal and increased over the duration of storage. The enhancement of water-holding capacity in yogurt resulted in a reduction of free water on its surface, consequently leading to a decrease in lightness and an increase in yellowness in PPP-fortified yogurts. Moreover, the yellow colour of PPP may result in reduced lightness and redness, alongside an increase in yellowness, as shown in the fortified yogurts. Yogurt colour changes during storage are likely due to acidity and structural alterations that result in diffusion of natural pigments into the yogurt matrix or surface (Turgut & Diler, 2025). Similar trends in colour changes have been documented after the incorporation of plant ingredients into yogurt and during the refrigeration of dairy products (Almusallam et al., 2021b).

**Table 2:** Colour attributes, colour changes (ΔE), and chroma (C*) of camel skim milk yogurt during storage prepared using microbial transglutaminase (MTGase) with and without pumpkin peel powder (PPP).

Treatment/Storage period (days)	Colour parameters				
	L*	a*	b*	ΔE	C*
*MTGase*					
1	86.72 ± 1.83^dC^	−1.98 ± 0.12^aA^	1.39 ± 0.65^bE^	–	2.42 ± 0.37^bE^
7	87.65 ± 0.35^cC^	−2.55 ± 0.04^bB^	3.67 ± 0.28^aD^	–	4.47 ± 0.45^aD^
14	95.45 ± 0.95^aA^	−2.42 ± 0.05^bB^	3.32 ± 0.02^aD^	–	4.11 ± 0.62^aD^
21	93.82 ± 0.52^bB^	−2.155 ± 0.09^bB^	3.57 ± 0.01^aD^	–	4.17 ± 0.39^aD^
*MTGase + PPP*					
1	83.75 ± 0.39^cF^	−2.51 ± 0.18^bB^	9.21 ± 0.02^cC^	8.38 ± 0.82^d^	9.54 ± 0.69^cC^
7	85.66 ± 1.11^aD^	−1.31 ± 0.07^aA^	12.64 ± 0.32^aA^	9.27 ± 0.77^c^	12.71 ± 0.85^aA^
14	84.77 ± 1.98^bE^	−1.95 ± 0.05^abA^	12.51 ± 0.37^aA^	11.43 ± 0.94^b^	12.66 ± 0.39^aA^
21	84.66 ± 0.15^bE^	−1.25 ± 0.11^aA^	10.35 ± 0.53^bB^	14.11 ± 1.02^a^	10.42 ± 0.42^bB^

Values are means of three samples ± SD. Small letters denote significant difference within treatment and big letters denote significant difference (P ≤ 0.05) between treatments.

### 3.6 Sensory evaluation of camel yogurt during cold storage

Table 3 displays the sensory parameters, including colour, taste, texture, sourness, and overall acceptability of MTGase-yogurt both with and without PPP. The incorporation of PPP into yogurt formulations exhibited varied effects on the sensory characteristics of the products. The colour was reduced; however, other quality parameters, including texture, flavor, sourness, and overall acceptability, were improved relative to MTGase-yogurt. The sensory property scores for yogurt containing PPP exceeded those of yogurt containing MTGase. The acceptability is likely attributable to the flavoring ingredients and high water-binding capacity of PPP. All sensory attributes improved with storage duration, with the lowest ratings for taste, flavor, and sourness recorded in MTGase-treated yogurt after 21 days, alongside a minor reduction in yogurt treated with PPP. The sensory quality scores for all yogurts remained above the threshold of 5 during the entire storage period. Our findings are consistent with prior research indicating that the addition of pomegranate juice powder (Pan et al., 2019), Argel leaf extract (Mohamed Ahmed et al., 2021), and date palm spikelet extract (Almusallam et al., 2021a) improves the sensory attributes of yogurts and maintains their quality during refrigeration. Abou-Soliman et al. (2020) demonstrated that the addition of MTGase to camel milk did not significantly alter the colour, flavor, or taste of cheese samples, whether untreated or treated with MTGase. Abou-Soliman et al. (2017) reported that MTGase-treated camel milk yogurts demonstrated average sensory attribute scores, acceptable appearance, overall acceptability, uniform structure, and no free water on the surface throughout the storage period. According to Bulca et al. (2022), the sensory attributes of yogurt produced from camel milk are improved with increased concentrations of MTGase.

**Table 3:** Sensory attributes of camel skim milk yogurt during storage, prepared using microbial transglutaminase (MTGase) with and without pumpkin peel powder (PPP).

Treatment/Storage period (days)	Sensory attributes					
	Colour	Texture	Taste	Flavor	Sourness	Overall acceptability
*MTGase*						
1	7.35 ± 0.81^aA^	6.31 ± 2.13^aB^	6.71 ± 2.27^aB^	6.52 ± 1.64^aB^	6.81 ± 1.79^aB^	6.67 ± 1.42^aB^
7	7.41 ± 1.19^aA^	6.45 ± 1.85^bB^	6.31 ± 2.27^aB^	6.61 ± 2.41^aB^	6.52 ± 1.88^bB^	6.65 ± 1.57^bB^
14	7.71 ± 1.13^aA^	6.59 ± 2.21^aB^	6.42 ± 2.03^aB^	6.75 ± 2.06^aB^	6.44 ± 1.68^aB^	6.41 ± 1.66^abB^
21	7.58 ± 1.32^aA^	5.63 ± 2.09^bB^	6.53 ± 2.14^aB^	6.63 ± 2.34^aB^	6.48 ± 2.06^bB^	6.58 ± 1.68^bB^
*MTGase + PPP*						
1	7.65 ± 1.05^aA^	7.51 ± 1.53^aA^	7.61 ± 1.01^aA^	7.82 ± 1.26^aA^	6.65 ± 1.08^aB^	6.44 ± 1.78^aB^
7	7.45 ± 1.86^aA^	6.65 ± 1.75^aB^	6.85 ± 1.59^aB^	6.65 ± 1.87^aB^	7.89 ± 1.82^bA^	6.87 ± 1.49^aB^
14	6.29 ± 1.21^bB^	7.66 ± 1.07^aA^	6.47 ± 1.84^aB^	7.07 ± 1.95^aB^	7.74 ± 1.75^bA^	6.96 ± 1.79^aB^
21	6.84 ± 1.54^bB^	7.31 ± 1.53^aA^	6.75 ± 1.31^aB^	6.87 ± 1.26^aB^	7.91 ± 1.59^abA^	7.54 ± 1.25^aA^

Values are means of three samples ± SD. Small letters denote significant difference within treatment and big letters denote significant difference (P ≤ 0.05) between treatments within the same storage period.

## 4 Conclusions

The current study suggests that incorporating MTGase into yogurt enhances its physicochemical and microbiological properties. The combination of PPP and MTGase further improved the physicochemical properties and microbiological stability of yogurts, presumably due to the antioxidant components present in PPP and the protein cross-linking activity facilitated by MTGase. PPP demonstrated stable microbial metabolic activity throughout 21 days of cold storage at 4 °C, maintaining high viable counts of both *S. thermophilus* and L. *bulgaricus*. Furthermore, it enhanced the total phenolic content (TPC) and DPPH activity of yogurt, suggesting that PPP may confer health benefits. The integration of PPP with MTGase enhanced the viscosity of yogurt during storage, resulting in improved consistency and appearance. The integration of PPP with MTGase enhanced customer acceptance of the product. The outcomes of this study may enhance the application of PPP in yogurt formulations, leading to improved storage stability and physical characteristics of the dairy product. This study is limited by the variability in peel coloration and the seasonal availability of pumpkins for production. The study could be expanded to investigate the mechanism underlying the MTGase effect on DPPH and to identify potential solutions to this problem without the inclusion of antioxidant sources.

## CRediT authorship contribution statement

**Rehab S. Alsulami:** Visualization, Methodology, Formal analysis, Data curation, Conceptualization. **Elfadil E. Babiker:** Writing – review & editing, Visualization, Validation, Supervision, Formal analysis, Conceptualization. **Isam A. Mohamed Ahmed:** Writing – original draft, Visualization, Validation, Software. **Tawfiq S. Alsulami:** Writing – review & editing, Validation, Supervision. **Hany M. Yehia:** Writing – review & editing, Visualization, Conceptualization. **Fahad Y. Al-Juhaimi:** Writing – review & editing, Visualization, Funding acquisition.

## Ethical approval

The study was conducted in accordance with the guidelines of the Declaration of Helsinki and was approved by the Institutional Review Board (IRB) of King Saud University, Kingdom of Saudi Arabia (Research Project No. E-25-9865).

## Funding

This work was supported and funded by Ongoing Research Funding program (ORF-2025-83), King Saud University, Riyadh, Saudi Arabia.

## Declaration of competing interest

The authors declare that they have no known competing financial interests or personal relationships that could have appeared to influence the work reported in this paper.

## Data Availability

The data supporting this study will be provided upon request.
